# Foveal light and dark adaptation in patients with glaucoma and healthy subjects: A case-control study

**DOI:** 10.1371/journal.pone.0193663

**Published:** 2018-03-06

**Authors:** Ronald A. J. M. Bierings, Marleen Kuiper, Casper M. van Berkel, Tom Overkempe, Nomdo M. Jansonius

**Affiliations:** Department of Ophthalmology, University of Groningen, University Medical Center Groningen, Groningen, the Netherlands; Justus Liebig Universitat Giessen, GERMANY

## Abstract

**Introduction:**

To determine whether foveal light and dark adaptation are affected in glaucoma.

**Methods:**

Case-control study with 23 glaucoma patients and 51 controls. Light and dark adaptation were measured twice. After 10 minutes pre-adaptation to 0.0032 cd/m2, the background luminance increased stepwise to 320 (5 log unit step) or 10,000 cd/m2 (6.5 log unit step) for 10 minutes, then it decreased back to 0.0032 cd/m2 for 30 minutes. Foveal contrast sensitivity [CS]) as a function of time was determined using a 1.15 degree increment. Time resolution of the experiments was 30 seconds. Multiple linear regression was used to analyse the effect of glaucoma on the CS plateau and adaptation time (time to reach the plateau minus 3 dB); analyses were adjusted for age and gender.

**Results:**

After light adaptation to 320 and 10,000 cd/m2, glaucoma patients had a 0.22 (P<0.001) and 0.13 (P = 0.010) log unit lower CS plateau than controls, respectively. After dark adaptation, this difference was 0.21 (P = 0.018) and 0.30 (P<0.001) log unit, respectively. Light adaptation occurred too fast to determine an accurate light adaptation time. Dark adaptation times of glaucoma patients and controls were similar, for both the 5 (7.2 versus 5.5 minutes; P = 0.10) and the 6.5 (18.2 versus 16.6 minutes; P = 0.14) log unit step.

**Conclusion:**

After a sudden increase or decrease in luminance, the logCS adaptation curves of glaucoma patients are shifted downwards compared to the curves of healthy subjects. Glaucoma patients have a lower CS plateau than healthy subjects, for both light and dark adaptation; dark adaptation times are similar.

## Introduction

Glaucoma is a chronic and progressive eye disease characterized by loss of retinal ganglion cells (RGCs) and subsequent loss of visual function. Traditionally, the loss of visual function has been described as asymptomatic, at least in early glaucoma [1]. However, asymptomatic seems to be the case only at appropriate luminance. Glaucoma patients, also those with early glaucoma, do complain regarding their visual performance under low, high, or changing luminance conditions [2–7]. So far, visual performance under changing luminance conditions is a largely unaddressed topic in glaucoma.

The most straightforward approach in exploring visual performance under changing luminance conditions is the measurement of the classical dark adaptation curve. Even though the rods and cones rather than the RGCs are the primary site where the visual system adapts itself to ambient luminance [8], impaired dark adaptation in glaucoma has been reported. The first studies that measured dark adaptation in glaucoma patients found a delayed curve for the central part [9–11] and the periphery of the visual field [12]. Variability, however, resulted in a poor diagnostic performance [13]. Others did not find clear differences in dark adaptation time between glaucoma patients and controls, neither for the peripheral visual field [14] nor for the central visual field [15], at odds with the earlier studies. Given the clear complaints emerging from the questionnaire studies, we considered a new, detailed look at this issue pivotal. Moreover, studies that measured light adaptation in glaucoma patients are apparently completely lacking.

The aim of this study was to determine whether foveal light and dark adaptation are affected in glaucoma. For this purpose we performed a case-control study involving glaucoma patients and healthy controls, all with a normal visual acuity. Following a paradigm as used by Zihl and Kerkhoff in brain-damaged patients [16], we measured Weber contrast sensitivity (CS) using a 1 degree diameter increment in the central visual field, after a stepwise increase or decrease in background luminance. We employed two step sizes, corresponding to respectively a dark environment versus a well-illuminated indoor setting and a dark environment versus outdoor at noon on a sunny day.

## Materials and methods

### Study population

In this prospective case-control study we included 23 glaucoma patients (cases) and two groups of 51 and 52 healthy subjects, respectively (controls). The ethics board of the University Medical Center Groningen (UMCG) approved the study protocol. All participants provided written informed consent. The study followed the tenets of the Declaration of Helsinki.

Glaucoma patients were selected from visitors of the outpatient department of the department of Ophthalmology, University Medical Center Groningen, using the visual field database of the Groningen Longitudinal Glaucoma Study (GLGS). The GLGS is an observational cohort study performed in a clinical setting [17]. The subpopulation selected for this study comprised primary open angle glaucoma patients with a best-corrected visual acuity (BCVA) of 0.0 logMAR or better (up to 50 years of age) or 0.1 logMAR or better (above 50 years), in at least one eye. In case both eyes were eligible, the eye with the lower (more negative) standard automated perimetry mean deviation (MD) value was chosen.

Controls were recruited by advertisement (posters with a call for participation as healthy volunteer in eye research were placed in public buildings in the city of Groningen). We aimed for subjects between 40 and 75 years of age, approximately 15 subjects per decennium per control group. Potential controls who responded to the advertisement filled out a questionnaire to screen for any known eye abnormality or a positive family history of glaucoma (exclusion criteria). After this preselection, an ophthalmic examination was performed, which included a BCVA measurement, a non-contact intraocular pressure (IOP) measurement (TCT80; Topcon Medical Systems, Oakland, USA), a frequency doubling technology visual field test (FDT C20-1 screening mode; Carl Zeiss, Jena, Germany), and a fundus examination with the Optos ultra-widefield retinal imaging device (200TX; Optos, Marlborough, USA). Exclusion criteria were any known eye abnormality, a positive family history of glaucoma, a BCVA worse than 0.0 logMAR (up to 50 years of age) or 0.1 logMAR (above 50 years), an IOP above 21 mmHg, any reproducibly abnormal test location at P<0.01 on the FDT test result, a vertical cup-disc ratio above 0.7 [18], or any other fundus abnormality, as observed by an ophthalmologist [NJ] who evaluated the Optos images and all other available data. If both eyes were eligible, one eye was randomly chosen.

### Data collection

Before the adaptation tests were performed, the pupil diameter was measured at two different luminances, being 2 and 320 cd/m^2^. A circular stimulus with a diameter of 12° was projected on a monitor (Radiforce G21; EIZO) in darkness. The testing distance was 0.5 m and the subjects were instructed to fixate at the middle of the stimulus, with one eye occluded using an eyepatch. After two minutes, a picture of the eye was taken using an eye-tracker. Pupil size was calculated using the ratio between pupil and white-to-white distance, assuming a white-to-white distance of 12 mm [19].

Adaptation was tested monocularly. We measured foveal contrast sensitivity during adaptation to a high luminance, after a previous adaptation to a low luminance (light adaptation), and during adaptation to a low luminance, after previous adaptation to a high luminance (dark adaptation). Before the experiment, the subjects received explanation in a dimly lit room; no additional bleaching was performed. Light and dark adaptation were measured twice, with a luminance step of 5 log units, and a luminance step of 6.5 log units. The group of glaucoma patients performed both step sizes, on a separate day; the two control groups performed each only one of the step sizes. For the 5 log units luminance step size, a high-luminance black and white monitor (Radiforce G21; EIZO; maximum luminance 470 cd/m^2^) was used with a testing distance of 0.5 meter; for the 6.5 log units step size, a projector (P1387W; Acer; maximum luminance 16,000 cd/m^2^, white light by driving the R, G, and B channel identically) positioned at the rear of a see-through PVC projection screen was used with a testing distance of 0.3 meter. This resulted in viewing angles of 44 degrees horizontally by 34 degrees vertically for the first setup, and 50 by 33 degrees for the second setup. The low-luminance condition was obtained by a 1 log unit decrease in luminance of the screen combined with absorptive neutral density (ND) filters with an optical density of 4 (transmission 1*10^−4^; #65–817 and #65–822, Edmund Optics) for the 5 log unit step, and of 5.5 (transmission 1*10^−5.5^; #65–817, #65–819, and #65–822, Edmund Optics) for the 6.5 log unit step. During the test, the patient’s head rested in a chin-rest to maintain the testing distance. Both setups were driven by the Psychophysics Toolbox (PTB-3; Brainard, 1997; Pelli, 1997) with Octave (version 3.2.4; www.gnu.org/software/octave/) for Linux (Ubuntu 10.10).

In both experiments, the test started with a 10 minute adaptation to the low-luminance condition, with a background luminance of 0.0032 cd/m^2^. After that, the background luminance increased stepwise to the high-luminance condition, with a background luminance of 320 (5 log unit step) or 10,000 cd/m^2^ (6.5 log unit step). Starting directly after the change in luminance, the foveal light detection threshold was determined every 30 seconds, for 10 minutes in total (light adaptation). Hereafter, the background luminance decreased stepwise back to 0.0032 cd/m^2^. Again, the foveal light detection threshold was determined every 30 seconds, for 20 minutes after the 5 log unit step and 30 minutes after the 6.5 log units step (dark adaptation). The foveal light detection threshold was determined using an increment with a diameter of 1.15 degree and a duration of 500 ms [16]. A 4–2 dB staircase procedure was used to determine the threshold Weber contrast ([L_stimulus_-L_background_]/[L_background_]); CS was the inverse of this threshold. The initial contrast was 0.0016. In between the stimuli there was a random interval with a mean (SD) duration of 1.6 (0.4) seconds. During each threshold determination, a fixation target surrounded the center of the screen. This fixation target consisted of four squares of 0.2° size, located at the horizontal and vertical meridian at 2° eccentricity. The experiments were performed with optimal correction for the viewing distance. As we were primarily interested in differences in overall visual function between glaucoma patients and healthy subjects, no cycloplegia, mydriasis, or artificial pupil was used. Measurements were preceded by a short familiarization trial. Luminance levels were measured with a Minolta luminance meter with built-in photometric filter (LS-110; Minolta Camera Co. Ltd., Japan).

### Data analysis

The study population was described using nonparametric descriptive statistics (median with interquartile range [IQR]). Univariable comparisons between cases and controls were made with a Mann-Whitney test (continuous variables) or Chi-square test with Yates correction (proportions).

Especially in the beginning of the dark adaptation phase, subjects were not always able to see the stimulus, even not at the highest contrast that could be offered. The logCS at these time points was defined as -1.3 (0.2 less than the lowest logCS that could be measured). However, later on, after at least two time points at which the stimulus was seen, unseen stimuli were considered missing (excluded from analysis). To avoid the inclusion of false-positive responses, we also excluded logCS values that were higher than the controls’ logCS plateau plus 2.6 standard deviations (Chauvenet’s criterion) [20].

To compare foveal light and dark adaptation between glaucoma patients and controls, we plotted the CS as a function of time. Glaucoma patients and controls appeared to differ regarding age. To enable a meaningful graphical representation of the data, we entered the controls with a weight factor. The weight factor was calculated, per 5-year bin, by dividing the number of glaucoma patients by the number of controls. The age-weighted control group was only used in the graphs.

Per subject, we determined the CS plateau after light and dark adaptation by taking the median CS of the last four measurements in the high-luminance (after 8 minutes), and the low-luminance (after 18 and 28 minutes, for the 5 and 6.5 log unit luminance step, respectively) condition. We defined the ‘adaptation time’ by considering a moving time window consisting of four consecutive time points. As soon as the median logCS belonging to these four time points came within -3 dB from the CS plateau, we took as the adaptation time the time halfway the second and third time point. The CS plateaus and the adaptation times of the glaucoma patients and controls were compared using multiple linear regression, adjusted for age and gender. A P value of 0.05 or less was considered statistically significant.

## Results

Table 1 shows the general characteristics of the study population. The glaucoma patients were older than the controls; glaucoma patients and controls did not differ regarding gender. Most patients had moderate or severe glaucoma in the study eye, with a median (IQR) visual field MD of -13.7 (-18.6 to -10.8) dB.

**Table 1:** Characteristics of study population.

	Cases (n = 23)	Controls *5 log unit step* (n = 51)	P value	Controls *6.5 log unit step* (n = 52)	P value
Age (year; median [IQR])	69 (61 to 73)	57 (49 to 65)	<0.001	58 (49 to 66)	<0.001
Gender, female, n (%)	9 (39%)	26 (51%)	0.49	27 (52%)	0.44
Pupil diameter at 2 cd/m^2^ (mm; median [IQR])	4.0 (3.0 to 4.7)	5.1 (4.5 to 5.5)	0.001^*^	5.3 (4.7 to 5.8)	<0.001^†^
Pupil diameter at 320 cd/m^2^ (mm; median [IQR])	3.2 (2.5 to 3.7)	3.0 (2.8 to 3.3)	0.79^‡^	3.0 (2.7 to 3.4)	0.81^§^
Visual acuity (logMAR; median [IQR])	0.00 (-0.08 to 0.00)	-0.08 (-0.08 to 0.00)	0.001^||^	-0.08 (-0.08 to 0.00)	0.001^#^
Median (IQR) HFA MD (dB)	-13.7 (-18.6 to -10.8)	NA	NA	NA	NA

IQR = interquartile range; HFA MD = Humphrey Field Analyzer mean deviation; NA = not applicable; age-adjusted P values:

* = 0.003 (median 4.9 mm)

† = 0.002 (median 5.3 mm)

‡ = 0.59 (median 3.0)

§ = 0.98 (median 3.2 mm)

|| = 0.005 (median -0.08)

# = 0.014 (median -0.06).

**Fig 1:**
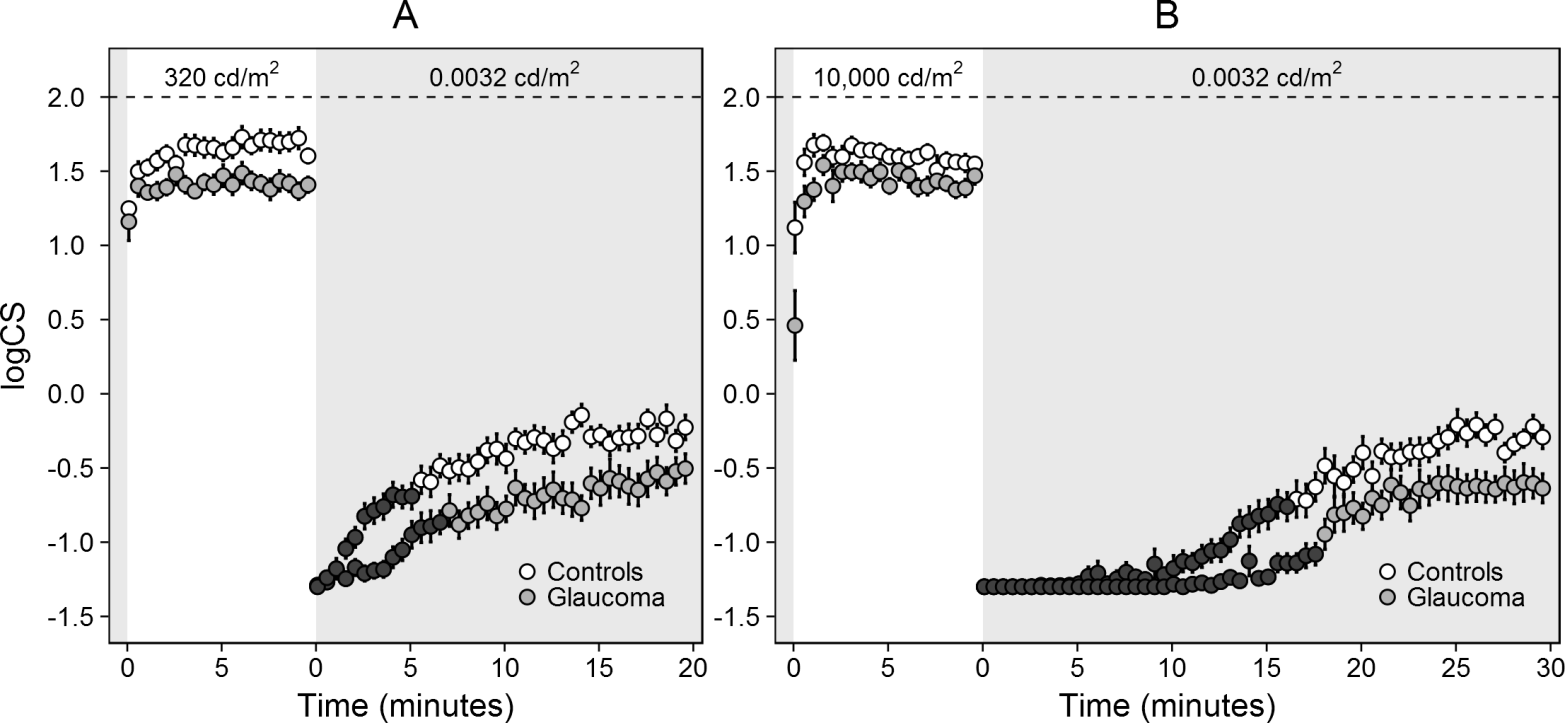
Contrast sensitivity (logCS) as a function of time for glaucoma patients (gray data points) and controls (white data points), for the 5 (A) and 6.5 (B) log unit change in luminance. Both tests were preceded by a 10 minute adaptation to a background luminance of 0.0032 cd/m^2^. The black data points correspond to a logCS more than 3 dB below the dark adaptation CS plateau (that is, the transition between the black and white/gray data points depicts the adaptation time). Error bars denote 1 standard error.

Fig 1 presents logCS as a function of time for the glaucoma patients and controls, for the 5 (Fig 1A) and 6.5 (Fig 1B) log unit luminance step. For the 5 log unit luminance step, the mean (SD) CS plateau after light adaptation was at logCS = 1.41 (0.27) for the glaucoma patients and at 1.66 (0.24) for the controls. After dark adaptation this was -0.58 (0.41) and -0.29 (-0.34). The mean (SD) dark adaptation time was 7.2 (4.7) and 5.5 (3.4) minutes for the glaucoma patients and the controls, respectively. Because both the glaucoma patients and the controls already reached their light adaptation CS plateau within the resolution of our sampling, a light adaptation time (see Methods section for definition) could not be determined. For the 6.5 log unit luminance step, the CS plateau after light adaptation was at logCS = 1.38 (0.23) for the glaucoma patients and at 1.55 (0.18) for the controls. After dark adaptation this was -0.63 (0.40) and -0.30 (0.30). The dark adaptation time was 18.2 (2.5) and 16.6 (4.5) minutes for the glaucoma patients and the controls, respectively.

Table 2 presents the corresponding multivariable analysis. For both luminance step sizes, the CS plateau after light and dark adaptation was lower in the glaucoma patients than in the controls. Dark adaptation time did not differ between glaucoma patients and controls.

**Table 2:** Multivariable regression analysis.

		*β*	P value
**5 log unit change in luminance (0.0032 versus 320 cd/m**^**2**^**)**			
Light adaptation CS plateau	Glaucoma^*^	-0.221	<0.001
	Age (years)	-0.010	<0.001
	Gender^†^	-0.130	0.020
Dark adaptation CS plateau	Glaucoma^*^	-0.214	0.018
	Age (years)	-0.015	0.005
	Gender^†^	-0.105	0.16
Dark adaptation time (minutes)	Glaucoma^*^	1.579	0.10
	Age (years)	0.121	0.006
	Gender^†^	1.091	0.17
**6.5 log unit change in luminance (0.0032 versus 10,000 cd/m**^**2**^**)**			
Light adaptation CS plateau	Glaucoma^*^	-0.134	0.010
	Age (years)	-0.004	0.038
	Gender^†^	0.030	0.49
Dark adaptation CS plateau	Glaucoma^*^	-0.297	<0.001
	Age (years)	-0.013	<0.001
	Gender^†^	-0.194	0.005
Dark adaptation time (minutes)	Glaucoma^*^	1.690	0.14
	Age (years)	0.127	0.011
	Gender^†^	-0.065	0.95

CS = contrast sensitivity; β = regression coefficient

* = glaucoma vs. controls

† = women vs. men.

For the subgroup of healthy subjects, the logCS of the dark adaptation plateau was significantly associated with age (*β* = -0.010 log unit per year for 0.0032 from 320 cd/m^2^ [P = 0.024]; *β* = -0.009 log unit per year for 0.0032 from 10,000 cd/m^2^ [P = 0.013]). The logCS of the light adaptation plateau was significantly associated with age at 320 cd/m^2^ (*β* = -0.009 log unit per year [P = 0.007]) but not at 10,000 cd/m^2^ (*β* = -0.003 log unit per year [P = 0.27]). All these analyses were adjusted for gender.

For the subgroup of glaucoma patients, the logCS of the dark and light adaptation plateaus were nonsignificantly associated with the visual field MD (*β* = 0.017 log unit per dB for 0.0032 from 320 cd/m^2^ [P = 0.19]; *β* = 0.015 log unit per dB for 0.0032 from 10,000 cd/m^2^ [P = 0.23]; *β* = 0.010 log unit per dB for 320 cd/m^2^ [P = 0.33]; *β* = 0.009 log unit per dB for 10,000 cd/m^2^ [P = 0.33]). All these analyses were adjusted for age and gender.

## Discussion

After a sudden increase or decrease in luminance, the logCS adaptation curves of glaucoma patients are shifted downwards compared to the curves of healthy subjects. Glaucoma patients have a lower CS plateau than healthy subjects, for both light and dark adaptation; dark adaptation times are similar.

Adaptation depends highly on testing conditions such as the luminance and time of pre-adaptation, the luminance to which a subject adapts, and the stimulus size and eccentricity [21–23]. The methods we used in our study were inspired by the experiment of Zihl and Kerkhoff, performed in healthy subjects and patients with brain damage. They also used a 1.15 degree, 500 ms foveal increment and a similar time structure to measure light and dark adaptation [16]. In contrast to our study, they used an asymmetrical design in terms of luminance: a pre-adaptation to 3.2 cd/m^2^, light adaptation to 320 cd/m^2^, and dark adaptation to 0.00032 cd/m^2^. We decided to make the luminance steps symmetrical, and thus made the pre-adaptation and dark adaptation luminance identical. The employed 0.0032 cd/m^2^ corresponds roughly to a starry sky without moon and is typically at the lower end of the luminance range that can be found outdoor in the public space after dark [24]. We adopted their 320 cd/m^2^ for light adaptation; we added a second experiment, with 10,000 cd/m^2^. In this way we mimicked both a well-illuminated indoor setting and outdoor at noon on a sunny day. Zihl and Kerkhoff found that almost all light adaptation happened within 2 minutes. This is in agreement with our findings. Baker studied light adaptation to 185 and 1850 cd/m^2^ from complete darkness (10 minutes), using a stimulus of 1 degree [25]. He found a similar pattern of light adaptation and-for 1850 cd/m^2^-also a small decrease in contrast sensitivity over time after approximately 3 minutes, similar to what we found for 10,000 cd/m^2^ (Fig 1B). Zihl and Kerkhoff reported a steady contrast sensitivity 12 minutes after a 6 log unit decrease in luminance. This accords with our adaptation times of 5.5 and 16.6 minutes after a 5 and 6.5 log unit decrease in luminance, respectively.

We did not find any study that measured light adaptation in glaucoma patients. Studies that measured dark adaptation in glaucoma patients mainly date back to the beginning of the previous century [9–12,14,26,27]. Generally, they found an impaired dark adaptation in glaucoma patients; differences in methodology, data reporting, case definition, and outcome measures inhibit a detailed quantitative comparison with our results. More recently, Jonas et al studied dark adaptation in glaucoma patients with a normal visual acuity, using a Goldmann-Weekers dark adaptometer (Haag-Streit, Berne, Switzerland) with a central stimulus of 11 degrees. In agreement with our findings, they found curves in glaucoma patients and age-matched controls that had a similar shape but differed in plateau [15]. Panos et al. found differences in dark adaptation between congenital and late-onset glaucoma; a direct comparison to healthy subjects was not reported [28].

We did not find a significant association between visual field MD and the logCS values of the dark and light adaptation plateaus. A possible explanation for this nonsignificance is the limited variability in MD in our patient group. However, all four *β* values were in the expected direction (positive, that is, a lower logCS with a more negative MD). Interestingly, if we multiply the *β* values (ranging from 0.009 to 0.017 log unit per dB; Results section) with the median MD of the glaucoma patients (-14 dB; Table 1), we get an answer close to -0.2 log unit, i.e., the loss of logCS attributed to glaucoma (Table 2). This tentatively suggests that glaucoma patients with little or no visual field loss would have roughly normal dark and light adaptation plateaus.

Intriguingly, three out of four CS plateaus were significantly lower in women (Table 2), which could not be explained by a gender difference in glaucoma severity or age (P = 0.42). Gender differences in CS have been reported before [29,30], and are consistent with a more pronounced visual illness perception in women than in men with glaucoma [5]. The decrease in CS with increasing age found in our study matches with results observed in clinical and population-based studies [31–34].

In this study, there was a difference in age distribution between glaucoma patients and controls. We initially included participants between 40 and 75 and aimed for a uniform age distribution. However, since glaucoma is a disease of the elderly, the vast majority of patients with glaucoma within our database was above 60 years of age. This made us recruit additional elderly controls. Nevertheless, a difference in age distribution between the groups remained. The distributions showed considerable overlap and all statistical analyses and graphs were adjusted for age. Therefore, this difference will not have influenced our findings. Albeit not intentionally matched, glaucoma patients and controls did not differ regarding gender (Table 1). Within the glaucoma group, the age distribution did not differ between male and female (P = 0.7). This was also the case within the control groups (both P = 0.6). As such, there was no collinearity between age and gender in our analysis.

The stimulus used in our experiments was a 1.15 degrees increment presented centrally. Therefore, we assumed to measure primarily cone function. However, the time that was needed to reach the CS plateau after the 6.5 log unit decrease in luminance appeared to be over 20 minutes in the healthy subjects. This suggests some rod involvement as well [21]. A possible explanation for the influence of rods in our experiment could be a less precise fixation during the dark adaptation phase (the fixation target was, despite its high contrast, barely visible especially during the beginning of the dark adaptation phase). In any case, glaucoma patients and healthy controls were susceptible to the same experimental conditions, and the adaptation differences between both groups appeared to be quite consistent. This is the first study that measured light adaptation in glaucoma patients, and focussed on the foveal part of the glaucomatous retina during dark adaption. Another strength is the unpreceded high luminance of 10,000 cd/m^2^ in the second experiment.

No cycloplegia, mydriasis, or artificial pupil was used. An advantage of this approach is that it gives insight in differences in the overall light and dark adaptation performance between glaucoma patients and healthy subjects, as the pupil reflex is one of the mechanisms contributing to adaptation. Another advantage is that it gives a more realistic insight in visual impairment. A clear drawback is that it is more difficult to study the glaucomatous changes in retinal sensitivity. At 320 cd/m^2^, the pupil diameter did not differ between the glaucoma patients and the controls (with and without adjustment for age; Table 1). Hence, the observed difference in light adaptation CS plateau at this luminance cannot be explained by a difference in pupil diameter and could thus be attributed to a difference in retinal sensitivity. We did not measure the pupil diameter at 10,000 cd/m^2^. Presumably, a significant part of the observed difference in light adaptation CS plateau at this luminance is caused by a difference in retinal sensitivity as well. At 2 cd/m^2^, the pupil was smaller in the glaucoma patients than in the controls (with and without adjustment for age; Table 1), and this may imply a difference in pupil diameter at 0.0032 cd/m^2^. Due to the Stiles-Crawford effect, this difference is not relevant to cone adaptation (our primary target), but may play a role in the confounding rod adaptation (see previous paragraph).

The essentially constant offset between the logCS of glaucoma patients and the controls during light and dark adaptation indicates an intact light and dark adaptation mechanism in the strictest sense (rod and cone function) together with an impaired signal processing downstream in the retina and beyond. This is in agreement with the presumed pathophysiology of glaucoma but apparently disagrees with the results of questionnaire studies (see Introduction section), which uncovered clear differences in visual complaints between glaucoma patients and healthy subjects when going from light to dark or dark to light. For dark adaptation, this discrepancy might be explained by postulating that a certain minimum CS is needed for reasonable vision. When adapting to darkness, glaucoma patients need longer to reach this minimum CS, which might explain their complaints when going from light to dark (glaucoma patients and controls had a similar dark adaptation time, but this time was defined as the time needed to reach 50% (-3 dB) of the CS plateau; as glaucoma patients have a lower CS plateau than the controls, they need longer to reach a certain absolute CS value). For light adaptation, the resolution of our sampling (one threshold per 30 seconds) makes it impossible to conclude if something similar plays a role when going from dark to light.

In conclusion, in the apparently intact foveal part of the visual field, glaucoma patients suffer from a reduced contrast sensitivity that is essentially independent of their adaptational state. This indicates an intact function of the outer retina together with an impaired modulation transfer in a later stage. As a result, during dark adaptation glaucoma patients reach a certain CS later than healthy subjects, which might explain their complaints when going from light to dark. Experiments with a better temporal resolution are needed to fully understand the complaints of glaucoma patients when going from dark to light.

## Supporting information

S1 File S1-file-data-underlying-this-study.xls. Data underlying this study.
(XLS)
